# Towards climate resilient urban energy systems: a review

**DOI:** 10.1093/nsr/nwaa134

**Published:** 2020-06-15

**Authors:** Vahid M Nik, A T D Perera, Deliang Chen

**Affiliations:** Division of Building Physics, Department of Building and Environmental Technology, Lund University, Lund 223 63, Sweden; Division of Building Technology, Department of Architecture and Civil Engineering, Chalmers University of Technology, Gothenburg 412 58, Sweden; Institute for Future Environments (IFE), Queensland University of Technology (QUT), Brisbane 4000, Australia; Solar Energy and Building Physics Laboratory (LESO-PB), Ecole Polytechnique Fédérale de Lausanne (EPFL), Lausanne 1015, Switzerland; Urban Energy Systems Laboratory, Empa, Dubendorf 86000, Switzerland; Regional Climate Group, Department of Earth Sciences, University of Gothenburg, Gothenburg 405 30, Sweden

**Keywords:** climate resilience, climate change adaptation, urban energy systems, renewable energy, extreme events, decentralized generation

## Abstract

Climate change and increased urban population are two major concerns for society. Moving towards more sustainable energy solutions in the urban context by integrating renewable energy technologies supports decarbonizing the energy sector and climate change mitigation. A successful transition also needs adequate consideration of climate change including extreme events to ensure the reliable performance of energy systems in the long run. This review provides an overview of and insight into the progress achieved in the energy sector to adapt to climate change, focusing on the climate resilience of urban energy systems. The state-of-the-art methodology to assess impacts of climate change including extreme events and uncertainties on the design and performance of energy systems is described and discussed. Climate resilience is an emerging concept that is increasingly used to represent the durability and stable performance of energy systems against extreme climate events. However, it has not yet been adequately explored and widely used, as its definition has not been clearly articulated and assessment is mostly based on qualitative aspects. This study reveals that a major limitation in the state-of-the-art is the inadequacy of climate change adaptation approaches in designing and preparing urban energy systems to satisfactorily address plausible extreme climate events. Furthermore, the complexity of the climate and energy models and the mismatch between their temporal and spatial resolutions are the major limitations in linking these models. Therefore, few studies have focused on the design and operation of urban energy infrastructure in terms of climate resilience. Considering the occurrence of extreme climate events and increasing demand for implementing climate adaptation strategies, the study highlights the importance of improving energy system models to consider future climate variations including extreme events to identify climate resilient energy transition pathways.

## INTRODUCTION

According to the Paris Agreement, countries should act to keep the increase in global average temperature to well below 2°C above pre-industrial levels. In particular, reducing the consequent risks and impacts of climate change needs to limit the increase to 1.5°C [1]. The energy sector plays an extremely important role in this regard. At least four of the United Nation's Sustainable Development Goals (SDGs)—SDG3-good health and well-being, SDG7-affordable and clean energy, SDG11-sustainable cities and communities, and SDG13-climate action—are highly relevant to the energy and climate policies. Limiting the increase in global average temperature requires decarbonizing energy generation on a global scale between 2030 and 2050 and achieving significant negative emissions in the second half of the century [2].

Cities and urban areas play a significant role in the energy transition and the path towards sustainability. Urban areas are characterized by high-energy density and heterogeneity in their energy use profiles [3]. Almost two-thirds of the global primary energy use is attributed to urban areas accommodating 3.5 billion people (around 50% of the world's population [4]), which leads to 71% of global direct energy-related greenhouse gas (GHG) emissions [5,6]. The projected population growth in urban areas (68% of the world's population by 2050 [4]), together with climate change and economic growth, will place greater stress on vital resources, including energy [7]. Low carbon and resilient strategies are needed when preparing cities for a huge influx of people by 2050 [8]. We should increase energy generation through sustainable approaches to cover future demands and move towards the climate targets. This requires a notable change in the energy infrastructure, accommodating renewable energy technologies such as solar and wind.

Renewable generation and energy demand are highly affected by climate conditions [9,10]. Climate change can induce intensified climate variations and consequently stronger and more frequent extreme events [11]. The frequency of some extreme events have increased over the last 30 years [12] and more weather-related disasters are expected in the future [13]. For example, in Europe increases are expected in heatwaves with shorter return periods, droughts, wildfires, river and coastal floods, and windstorms [14]. Such extreme conditions can affect two-thirds of the European population by 2100 [15]. One example is the 2003 heatwave in Europe which caused 70 000 excess summer deaths as a result of, among other factors, maladapted built environments [16]. Climate-induced risks, many resulting from extreme climate events [17], should be recognized before the further development of energy infrastructures [18]. Extreme weather events are one of the main reasons for energy disturbances [19]. The annual cost of blackouts from extreme weather conditions ranges from $20 to $55 billion in the USA [20]. Inadequate climate-focused strategies can affect energy security and induce extra costs. Rather than just aiming for decarbonizing the energy systems and climate change mitigation, it is essential to plan for climate change adaptation as well, especially in urban areas with complex energy flows and interactions. Climate change mitigation and adaptation strategies should be assessed in parallel, considering a wide range of actions from supply to demand. This requires a reliable impact assessment of climate change on energy systems and proper evaluation of sustainable energy solutions.

Understanding the impacts of climate variations on the energy infrastructure is extremely challenging because of the multivariate and multiscale changes of the climate system [21] as well as the complex workflows between climate models and energy systems. Failing to address these challenges can lead to significant performance drops in the energy systems. Furthermore, uncertainties brought by climate change can easily lead to blackouts especially considering high renewable energy penetration levels [10]. Therefore, improving the energy systems to withstand these climate variations in a robust and resilient manner is vital to make the sustainable energy transition a reality. Enhancing the connectivity between climate and energy system models and improving the design of energy systems to withstand future climate variations in a flexible manner will play a vital role in the sustainable transition of energy systems. There has been significant progress in developing climate models and projecting future climate conditions over the last two decades. Moreover, in the recent past, energy models have been developed to consider uncertainties at the design and operation phases including many complex interactions among different actors within the energy domain. Linking these models to properly understand and quantify the impacts of climate change on the energy system brings unprecedented opportunities to assess and improve the design and performance of energy systems. However, there exist limitations in this regard, which makes the transition of energy systems challenging. This paper investigates this research gap by focusing on climate resilience [22] of urban energy systems [10]. Bridging this gap enables us to enhance the connectivity and assess the impacts of climate change by addressing extreme conditions, assessing risks, analysing possible remedies and drawing promising pathways to the energy system transition.

The concept of resilience within the energy system domain is complex and multi-faceted [19] and can be used to address extraordinary conditions with different natures. However, most of the available works are focused on detecting faults in the energy supply networks, dealing with spatial scales much larger than the city scale and not considering the complexities in urban areas [23]. Climate resilience is an emerging concept that is increasingly used to represent the durability and stable performance of energy systems against extreme climate events. Despite the growing interest in climate resilience, the concept has not yet been adequately explored in relation to the recent advances in climate change modelling [17].

This work summarizes studies on climate resilience of energy systems and promising methods to incorporate climate resilience into the energy system design and assessment process in urban areas based on the present state-of-the-art. An overview of the transition of urban energy systems is provided, which also discusses the upcoming challenges and expectations. The next section provides a review of the impacts of climate change on different aspects of energy systems and urban energy conditions. Then, there is discussion of the concept of resilience and its interpretation in the context of energy studies. An explanation is provided of the major computational challenges in the assessment of energy systems for future climatic conditions. The penultimate section suggests some pathways to assess the climate resilience of urban energy systems considering future climate models. Finally, the concluding remarks are presented.

## TRANSITION OF URBAN ENERGY SYSTEMS AND CHALLENGES ASSOCIATED WITH THEIR CLIMATE CHANGE ADAPTATION

The Fifth Assessment Report (AR5) of the Intergovernmental Panel on Climate Change (IPCC) defines an energy system as ‘all components related to the production, conversion, delivery, and use of energy’ [24]. An energy system is a socio-technical system that handles the combined processes of acquiring and using energy in a given society or economy, with strong connections to markets, consumer behaviours and other factors that affect the construction and operation of the technical infrastructures [25]. Urban energy systems are those designed to cater the energy demand in cities and urban areas, and they gain from having an optimal scale for combining energy conservation programs with promising energy strategies [26]. An urban environment is usually separated from many of the energy system-related processes, such as extraction of resources, refining, transportation and storage. Therefore, adapting the definition of an energy system to an urban energy system depends on how the boundaries of a city are defined. There are arguably three definitions: 1) pure geographic, which considers the technologies that lie within a city's administrative boundaries; 2) geographic-plus, which adds the traceable upstream flows to the pure geographic definition; 3) pure consumption, which encompasses all the energy activities of a city's occupants wherever they occur [25]. The perspective that we have in this work is closer to the second definition. Urban energy systems go through transition to improve their efficiency and sustainability. Not all the elements related to the transition, such as introduction of block chains, energy informatics, intelligent grid, machine learning, are related to the scope of this work and our main focus is on the elements that are related to the urban climate.

The classical decentralized power systems based on fossil fuel sources and the associated grid (electricity, heat and gas) possess the capability to withstand the variations in the energy demand without a considerable impact on the reliability of the energy supply. In a world longing for renewable energy as a result of climate, social and health (e.g. caused by photochemical smog in cities) concerns as well as the depletion of fossil fuel resources and uncertainties in the energy market, major changes are happening in the energy infrastructure [27,28]. However, moving towards renewable energy generation induces several challenges. For example, power generation by solar photovoltaic (PV) and wind turbine technologies is strongly influenced by weather conditions. Often the peak energy demand does not follow the peak generation and catering the mismatch requires auxiliary solutions such as energy storage, fossil fuel or nuclear generators. Enabling energy storage devices to work in harmony with the existing infrastructure [28] is very challenging, meanwhile vital, for the sustainable energy transition. Energy management becomes more challenging when integrating renewable generation, and more critical to guarantee grid stability. Renewable energy generation is mostly based on distributed energy sources, unlike non-renewable sources such as fossil fuel and nuclear energy. This can change the roles and relations in the energy market, for example, the large-scale deployment of building-integrated PV technology can change the classical consumers to prosumers who both produce and use energy [29,30]. This alters the one-way flow of energy from the producer to the consumer (also called radial flow), and consequently transforms the role of energy grids dramatically. Accommodating the changes in the system design and grid plays a vital role in the energy transition, which can be considered as a major challenge.

Besides the structural changes that require accommodating renewable energy technologies, energy infrastructure is strongly influenced by changes that take place in the transportation and building sectors, especially in cities and urban areas [31]. The transport sector is responsible for 15% of CO_2_ emissions [32], which contributes considerably to climate change. The electrification of the transport sector is highly promoted nowadays as a sustainable solution, especially in urban areas. This notably influences the net energy demand as well as the demand pattern. Similarly, the building sector brings many challenges into the grid [33], for example increasing the urban density and consequently higher demands and peaks. Inclusion of different sectors, such as transportation and district heating/cooling, and developing an integrated infrastructure enable the decarbonization of multiple sectors at the same time [34]. However, the design and operation of such an interconnected infrastructure is a major challenge. Malfunctions in one sector can easily penetrate the whole infrastructure, leading to blackouts especially during extreme conditions such as extreme climate events [10]. These all indicate that the energy sector is becoming the backbone of urban infrastructures, which brings many challenges especially concerning its transition [34].

In addition to the altered flow of energy and having prosumers affecting the energy grid, energy storage is getting popular at the domestic scale to profit from the fluctuations in real-time pricing [29], which will be driven by cyber interactions among different parties. This can play a critical role in boosting the resilience of energy systems, usually by empowering the autonomous operation of the energy system, especially in the form of distributed energy storage in smart grids, also known as community energy storage (CES) [30,35]. Smart heating, Vehicle to Grid (V2G), etc., are also used to take advantage of the fluctuating energy demand and generation. The operation of prosumers, distributed system operators, ancillary grid service providers, etc., strongly depend on the information flow between different parties [36]. For example, distributed system operators would be eager to know the future forecast of energy demand while prosumers would be more

interested in the forecast of spot price (peer-to-peer market). Information flow between different parties can play a critical role in this regard especially in linking different sectors with the energy infrastructure (also known as sector coupling). This can convert urban energy systems into a cyber-physical system [37]. Further, this can also extend the smart grid concept and open windows to the energy internet. The emergence of cyber-physical interactions between different parties within the energy domain is considered as the next challenge.

**Figure 1.**
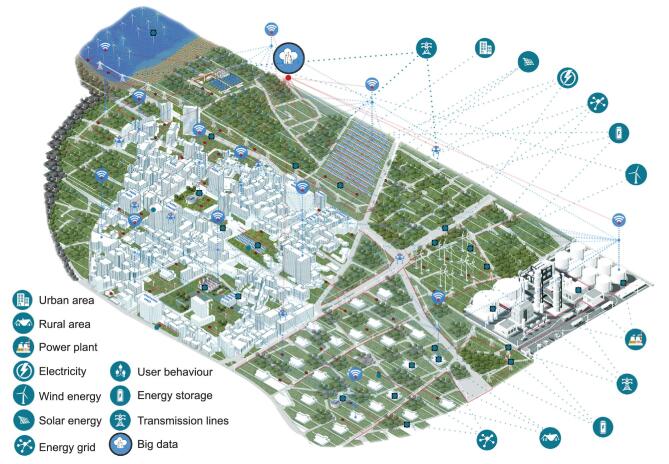
An urban area is a multi-complex system with strong interactions. Climate is a major factor, affecting the energy demand, renewable generation and citizens’ comfort. Urban climate and its spatial and temporal variations, induced by natural variations and/or urban morphology, are the boundary conditions that affect the urban systems, buildings and citizens. Urban energy systems, as multi-variant cyber-physical systems, have complex interactions with their environments. The complexity increases by the higher integration of decentralized renewable energy generation, making the energy flow more complex, compared to a centralized system (e.g. supplied by a power plant). Climate change and intensified weather variations can challenge the energy systems and consequently living conditions in urban areas. Becoming climate resilient demands further learning more about the complex interactions, taking advantage of data science and technology enhancement and counting for extreme events.

Climate change will introduce many changes to energy systems, affecting different aspects of the energy flow from generation to demand [18]. Most of these changes lead to considerable uncertainties in the energy infrastructure at different levels, for example uncertainties in equipment, inputs and outputs [38]. The availability of renewable energy, for example wind and solar energy, is an important input that depends on climate conditions. Output uncertainties are affected by demand conditions and uncertainties in building parameters, occupant behaviour, climate conditions and control strategies [38,39]. The demand side uncertainties can get intensified in urban areas because of increased complexity, covering a wide range of concepts and disciplines, for example from building physics to social psychology [40]. Climate change and its uncertainties can affect the demand and generation sides considerably [9], with significant impacts on the transition of energy systems [10] (see Fig. 1). At one end, climate change mitigation pushes to bring up many changes into the energy systems while on the other side extreme climate events and non-extreme climate variations make the climate change adaptation and energy transition more challenging. This creates a vicious cycle. Improving the flexibility and climate resilience of energy systems is the way to cope with climate change and extreme events, which is vital for a reliable energy transition.

## IMPACTS OF CLIMATE CHANGE INCLUDING EXTREME EVENTS ON ENERGY SYSTEMS

Climate change entails the temporal and spatial variations of weather conditions and their statistics, inducing deviations from the common patterns of energy demand and generation. Such variations come with large uncertainties and most probably strong extreme events that make the energy system deviate from the generally accepted guidelines. Extreme climatic events and uncertainties can induce increased risks at all levels within the energy sector, including design and operation [18]. Impacts of climate change on the energy system can be roughly classified into three categories including energy infrastructures, energy supply and energy demand [41,42], affecting different aspects of urban energy conditions. Ciscar and Dowling [41] briefly reviewed the approaches for impact assessment of climate change on the energy sector, considering qualitative economic and energy models with top-down (economic) and bottom-up (engineering or techno-economic) approaches. Table 1 summarizes some recent studies about the impact assessment of climate change on the energy systems.

**Table 1.** An overview of some research works about the impact assessment of climate change on energy systems.

			Impact of climate change on					
Reference	Considered uncertainty	Extreme climate	Demand	Generation	Temporal resolution	Methodology used to consider uncertainty	Criterions for the assessment	Climate model used
[43–46]	Climate	Considered	Heating and cooling	Considered for solar and wind	Hourly	Deterministic, stochastic and robust techniques in system optimization	Cost, autonomy, CO_2_ emissions, utilization of renewable energy	RCM and urban climate
[47,48]	Climate	No	Considered	Considered	Hourly	Deterministic, stochastic and robust techniques in system optimization	Cost and CO_2_ emissions	RCM
[49]	Climate	Considered	Considered	Considered	Three hour	Mixed integer linear problem used and climate uncertainty considered by using sensitivity analysis	Cost	RCM
[50]	Price and policy	No	No	No	Hourly	Stochastic	Cost	No
[51]	Climate	No	No	Considered for solar, wind and hydro	Three segments per year	Considered by using chance constrained method	Cost	No
[52]	Climate	No	No	Considered through a global sensitivity analysis	Yearly simulation	Stochastic model	Cost	No
[53]	Climate	No	Considered	Considered	Monthly simulation	Combining Monte Carlo simulation with a deterministic model	Cost	No
[54]	Climate	No	Considered	No	Yearly simulation	Duel possibility-based approach	Cost	No
[10]	Climate and prices	Yes	Heating and cooling	Considered	Hourly	Stochastic-robust	Cost and system autonomy	GCM and RCM

Some of the climate-induced problems for the energy infrastructure result from short-term extreme events (short-duration events such as heat-induced sagging and hurricanes) as well as long-term changes in climate (e.g. drought and water shortage). Harsh extremes and frequent variations (fluctuations) can induce shocks to the system and push the performance limits of its components. Despite the low probability of extreme weather conditions, their consequences can be very costly. Around 80% of the large-scale power disruptions during 2003–2012 in the United States were induced by extreme weather [12]. Most of the available literature is about the failures in the energy transmission networks from extreme events such as tornados, focusing on power plants, transmission and grid infrastructure. Power supply and system operation can get disturbed by several types of extreme weather conditions, such as high temperatures and winds, heavy snow and floods [55]. Oil and gas supply can be disrupted by extreme weather events too, leading to production shutdowns, such as hurricanes in the Gulf of Mexico in 2004 and 2005 [56]. The increased average temperature can result in longer warm seasons and an increased probability of wildfires, posing a threat to power transmission facilities [19]. There also will be some secondary effects, for example reduced efficiency of energy system components from high water temperature, which affects the cooling of power plants [56].

The availability of renewable energy which depends on climate conditions is an important factor for the performance of distributed energy systems (DES). It is expected that the impacts of climate change become more severe with increased integration of renewable generation into energy systems [42], as studied for wind [57], hydropower [58] and solar energy [59]. The scale of the impact depends on the renewable source and geographical location. For example, several researchers have shown that the evolution of wind from climate change is quite weak for the twenty-first century [57,60]; however, climate uncertainties can affect the assessment considerably [61]. Very few studies have looked into the impacts of climate change on solar energy potentials, reporting large uncertainties in the cloud cover estimations induced by global climate models (GCMs) [59]. Impacts of climate change on solar energy depend on the geographical location, for example it may increase solar potentials for south-eastern Europe [62] and decrease for Canada [56]. The secondary impacts of climate change may also affect solar energy generation, for instance by reducing the efficiency of photovoltaic cells because of higher temperatures [56]. Energy generation can shift more towards water-dependent technologies in the future, which implies that a significant amount of uncertainty will be added to the already uncertain operation of hydropower systems from climate change [56], for example the uncertainty range of ±9 TWh in estimating the hydropower energy generation per year [58]. Some other impacts of climate change on the energy supply can be the lower efficiency of thermoelectric and nuclear plant generation through lower availability of cooling water, lower availability of biomass from less production, less hydropower resources and lower efficiency of renewable electricity technologies [18,42]. It is interesting to know that the major climate change-induced challenges for solar and wind energy generation are realized at fine temporal resolutions [63], which are often not captured.

In addition to assessing the availability of renewable energy generation, proper adaptation of energy systems also requires reasonable estimations for the amount and variations of energy demand in the future, with suitable temporal and spatial resolutions. There are some works assessing the effects of climate change on the energy demand in urban areas at different spatial scales such as city [64], country [65,66] and even continent [67], mostly focusing on buildings as the major components of urban areas. Climate uncertainties can affect considerably estimations of the energy demand, as shown by Nik [64] through implementation of several future climate scenarios, based on the 4th Assessment Report (AR4) of IPCC and with different spatial resolutions, simulating the energy demand of the Swedish building stock. The adopted temporal resolution can also affect the assessment results considerably. For example, for the case of assessing the energy demand of the residential building stock in Stockholm, the hourly heating and cooling demand reached, respectively, 50% and 400% above the typical conditions during extreme climatic conditions [64]. This illustrates the critical situation for energy systems to cover peak demands in future [68]. The uncertainties from the demand side can be amplified in urban areas because of the increased complexity, covering a wide range of concepts and disciplines, for example from building physics to social psychology [40]. Nik [9] reviewed some major works that study the impacts of climate change on buildings and their energy performance. In general, impacts of climate change and extreme weather events on the peak demand are well beyond the net annual change in energy demand. This makes it challenging at both the design and operation phases of urban energy systems, requiring improvement of the flexibility and robustness of energy systems to withstand climate variations and extremes. Hence we should assess the impacts of climate change at both the design and operation stages where proper quantification of climate resilience plays a major role [10].

## RESILIENCE OF ENERGY SYSTEMS: STATE-OF-THE-ART

The concept of climate resilience is introduced corresponding to situations that a system can function during (and/or after) extreme climate events, e.g. Ref. [22]. A resilient system should be able to respond to change and bounce back towards equilibrium or stability after an extreme event [69]. There is no standard definition for the resilience of energy systems [70] and it varies, depending on the context and objective. In general, a resilient energy system should speedily recover and learn from shocks and provide alternative means of satisfying energy service needs [71,72]. Resilience measures can be divided into two groups of short-term and long-term measures, with the former referring to preventive and corrective actions and the latter to planning for climate change adaptation [19]. Table 2 presents some definitions of resilience in the literature focusing on the climate resilience of energy systems.

**Table 2.** Resilience definitions with the focus on energy systems.

Resilience is defined as the ability of an energy system to:	Reference
absorb, adapt and respond to changes	[73]
respond to change and getting back to equilibrium or stability	[69]
avoid or minimize interruptions of service during an extraordinary and hazardous event	[74]
plan for, recover from and adapt to adverse events over time	[75]
tolerate disturbance and continue delivering affordable energy services to consumers	[71]
have a secure energy supply chain that withstands shocks and adopts (flexibility + elasticity)	[76]
anticipate, absorb, adapt to and/or rapidly recover from a disruptive event	[77]
withstand extraordinary events with high impact and low probability; recover fast after disruption; learn to adapt and prevent/mitigate similar impacts	[19,78]
reorganize during perturbations, with the assistance of new states through using different processes and structures	[79]
maintain reliability at extreme events	[80,81]
meet performance levels as it is in normal operation during a disruption	[82]
recover to pre-disturbance secure state after the gradual degradation under increasing system stress	[83]

**Figure 2.**
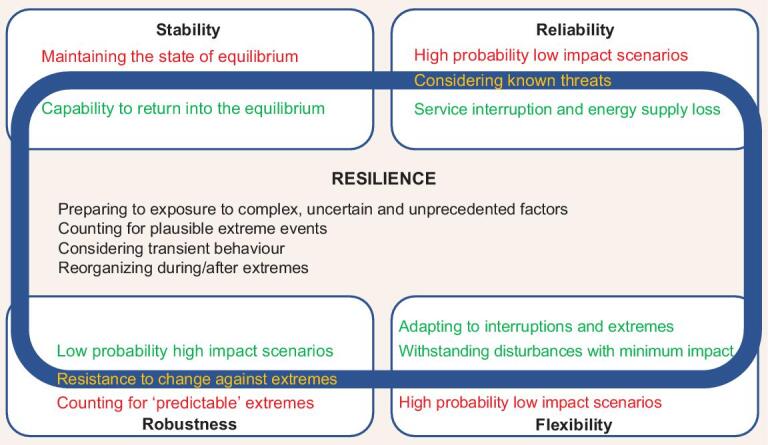
The specific characteristics of resilience (text in black) and its similarities (text in green and yellow) and differences (text in red) with stability, reliability, robustness and flexibility. The yellow texts on the border of resilience are not always considered in studying resilience.

Research works about the resilience of energy systems gain from the earlier works on the ‘reliability’ of power systems, such as Ref. [84]. The reliability-oriented approach is mainly focused on the known threats, while the resilience-oriented approach also counts for extremes that may have not been experienced before Ref. [70]. The main difference between the two approaches is that reliability often relates to ‘high probability low impact’ scenarios (which does not consider extremes), while resilience relates to ‘low probability high impact’ scenarios [85]. This is also the difference between ‘flexibility’ and resilience [86,87]. Resilience can be closely associated with ‘robustness’ and ‘stability’, which are often used in control systems with wide applicability in power system engineering. Robustness mostly refers to the resistance to change against extreme conditions, while resilience puts an emphasis on flexibility and survivability [88]. A robust energy system can be secured by accounting for ‘predictable’ natural and technical factors followed by upgrading infrastructure and switching to more abundant resources. On the other hand, resilience is attained when the energy system is prepared for diverse and partially ‘unpredictable’ factors by increasing its ability to withstand and recover from various disruptions. This has to do with the fact that resilience reflects and provides ways to deal with generic concerns arising from exposure to complex and uncertain factors. Stability is defined as the capability of returning to the original or desirable equilibrium following a disruption (the sooner the return, the better the stability) [89]. However, the concept of stability does not consider the continuous evolution of the system [90], which is also a limitation in the concept of reliability [80]. The aforementioned concepts are summarized in Table 3 and reflected in Fig. 2.

**Table 3.** Some concepts that describe the energy system in relation to a shock or an extraordinary event.

Concept	Definition
Reliability	Addresses the issues of service interruption and energy supply loss Relates to high probability low impact scenarios Focuses on known threats Does not consider the continuous evolution of the system
Stability	Addresses the capability to return into the equilibrium following a disruption Mostly defined as maintaining the state of equilibrium Does not consider the continuous evolution of the system
Robustness	Addresses the resistance to change against interruptions/extremes Relates to low probability high impact scenarios Can be secured by counting for ‘predictable’ natural and technical factors
Flexibility	Addresses the capability to modify generation/consumption patterns in reaction to interruptions/extremes Mostly defined as the capability of the system to withstand the external disturbances with a minimum impact on its performance Relates to high probability low impact scenarios
Resilience	Addresses the flexibility and survivability against extremes Reflects concerns arising from exposure to complex and uncertain factors Relates to low probability high impact scenarios Counts for extremes that may have not been experienced before Prepares for diverse and partially ‘unpredictable’ factors Considers transient behaviour and measures the capability of a system to reorganize during/after the perturbations

**Figure 3.**
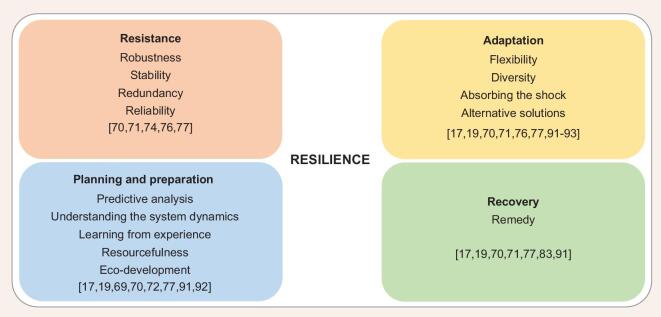
Dividing the resilience characteristics into four major groups based on resisting, adapting to, preparing for and recovering from an extraordinary event.

Characterizing climate resilience is highly dependent on the considered infrastructure, phenomena, climate-induced risks, as well as spatial and temporal scales. General definitions for resilience such as the ‘ability to anticipate, absorb, adapt to and/or rapidly recover from a disruptive event’ [77] provide enough space to select and/or define the characteristics and performance criteria that match the purpose of the assessment. Despite the considered characteristics, they should facilitate to avoid or minimize interruptions of service during extraordinary events. Figure 3 summarizes the major characteristics that are counted in the literature for a resilient energy system. Based on their nature, these characteristics can be divided into four major groups of (1) resisting, (2) adapting to, (3) preparing for and (4) recovering from an extraordinary event. A resilient energy system should have a combination of these characteristics with different natures. For example, a combination of robustness, resourcefulness, recovery and adaptability [19,70,91] or a combination of resistance, reliability, redundancy and recovery [77]. However, most of these concepts have been articulated without considering climate change and future climate modelling. Therefore, it is expected to see modifications in such characterizations in the near future.

Resilience thinking can be interpreted as an approach to the management of socio-ecological systems aiming to develop an integrated framework for disaster risk management [17]. The frameworks for assessing and managing resilience are mainly inspired by the ones used for risk assessment and take risk analysis as a central component [75]. But the resilience frameworks go beyond the boundaries of risk management ‘to address the complexities of large integrated systems and the uncertainty of future threats, especially those associated with climate change’ [75]. Planning for resilience requires the ability to predict the future and to understand the governing system dynamics. Depending on the research scope and case studies, there are differences in definitions, characteristics and assessment criteria that are used in the framework. As it is challenging to quantify resilience from the energy system perspective, it is also interpreted as an extension of the power supply reliability [74]. Table 4 provides an overview of the major approaches and methods used for assessing the resilience of energy systems. According to Panteli and Mancarella [19], a proper research framework should have three main models for weather, component and system, aiming at the quantitative assessment of climate impacts on the key resilience features. Considering power systems, the modelling techniques are divided into two major groups of analytical and simulations techniques. The advantage of analytical techniques is their relative simplicity; however, simulations are recommended for the resilience studies as the climate is a complex system with a variant and stochastic nature. Most of the simulation techniques for assessing resilience are based on Monte Carlo (MC) simulations. Concerning urban energy systems, a novel framework was designed and applied by Perera *et al*. [10] which enables quantification of climate resilience of urban energy systems considering climate uncertainties. The key to the climate resilience assessment is the proper linkage between climate and energy models. Besides considering climate uncertainties, it is important to adopt a suitable temporal resolution for the analyses to reveal the risk of extreme events. This allows counting for ‘unprecedented’ extreme events which are physically ‘plausible’ and reflected by future climate models. Some works refer to these events as ‘unforeseeable’ events (mainly inspired by risk assessment, e.g. Ref. [94]); however, for assessing ‘climate resilience’, unprecedented climate events are foreseeable using climate models, so we refer to these events as ‘plausible’ extreme events.

**Table 4.** Some major approaches for assessing the climate resilience of energy systems.

Approach	Reference
Measuring resilience considering the type of extreme event, time duration of the disruption event and its impact on the performance indicators	[82]
Formulating resilience simply as the capability of the system to maintain reliability during extreme events	[80,81]
System-of-systems: a framework with three main models for weather, component and system	[19]
Applying the risk management and investment perspective and using reliability assessment frameworks to deal with hazardous conditions	[74]
Determining the capacity of the system to fulfil energy security through considering relations and interactions between significant elements of the energy system	[71,76]
Classifying the disruptive climate-induced events into five categories of small, moderate, serious, major and extreme impact, depending on 1) the frequency and duration of the event, and 2) the number of customers being affected	[12]
Proposing a framework to evaluate energy security^a^ under long-term energy scenarios (generated by integrated assessment models)	[93]
Distinguishing three basic weather conditions: normal, adverse and extreme Assessing the weather-related faults: single and multiple Assessing physical damages and their effects (on the electricity supply and distribution network): ranging from ‘no effect’ till circuit outages and significant physical damage	[55]
Modelling the performance of the system as a sequence of random events, affecting each other by the time Introducing sequential Monte-Carlo-based time-series simulation model Using the concept of components’ fragility curves for applying weather- and time-dependent failure probabilities Dividing resilience into short- and long-term resilience as a function of time Planning short-term measures (preventive and corrective actions) with the use of weather forecasts in the scale of days/hours Planning for climate change adaptation and robustness to achieve long-term resilience by risk and reliability studies in which plausible future scenarios are considered	[70]
Considering multiple climate scenarios from regional climate models Generating extreme and non-extreme time series for energy demand and generation Stochastic robust optimization of the energy system Assessing indicators (cost and system autonomy)	[10]

^a^Energy security is defined as low vulnerability (treated as a combination of risks associated with inter-regional energy trade) of vital energy systems.

## CHALLENGES IN THE IMPACT ASSESSMENT OF CLIMATE CHANGE ON URBAN ENERGY SYSTEMS

**Figure 4.**
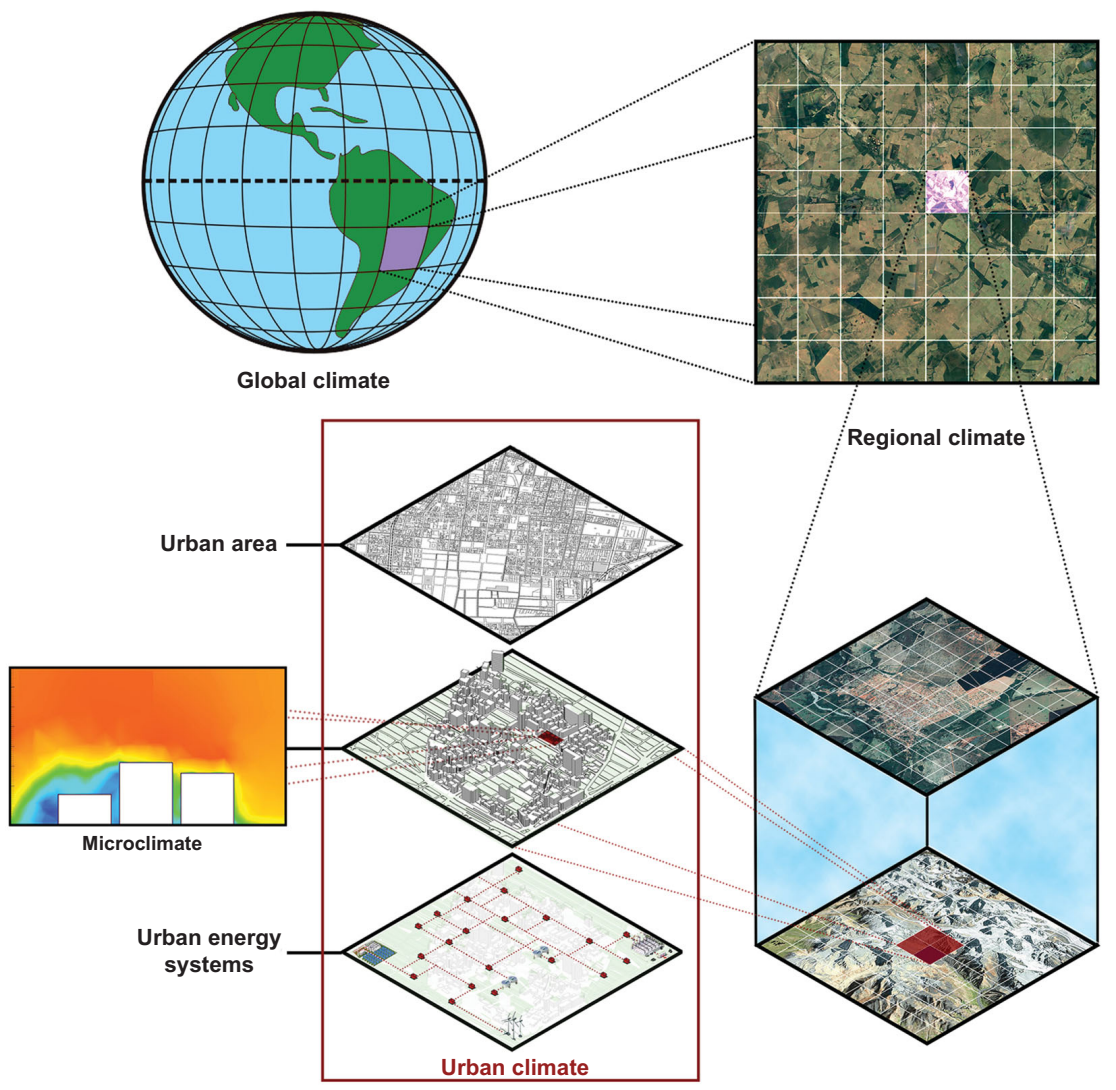
The energy performance of urban areas is tightly connected to the urban climate conditions. Variations in the global climate affect the regional and urban climate.

Climate is a very dynamic system and studying its behaviour depends highly on the selected temporal and spatial resolutions. The climate that affects citizens, buildings and energy systems in urban areas is urban climate, which is the altered version of the regional climate in a finer spatial scale and affected by the energy and material flows in the urban context. Figure 4 schematically explains the connection between urban energy conditions and climate. Variations in the global climate will be transferred to the urban climate (and even microclimate with much smaller spatial scales), affecting the performance of buildings and energy systems. Depending on the urban design and morphology, climate variations can get amplified or dampened in the urban scale [95,96]. Currently, the climate research community are focused on global and regional climate models (GCMs and RCMs), while the urban scale model is rare and not coordinated. An RCM (usually with spatial resolution of 20–50 km, although higher resolution modelling is becoming increasingly available) is usually nested in a GCM (with the spatial resolution of 100–300 km) and driven by the conditions of the global climate at the boundaries of the RCM domain. It is well known that RCMs can reproduce a more realistic regional climate, especially with regard to extremes [97].

To assess the impacts of climate change on energy systems, meteorological data for the past/present (baseline or reference) and future climate are needed. While future climate information can only be provided by GCM and/or RCM, the past/present data climate can be represented by historical observations or GCM/RCM simulation for the historical climate. A common approach in energy studies is to use a one-year typical weather data set to represent climate over a 30-year period, known to be a Typical Meteorological Year (TMY). TMY helps to represent typical conditions for the past/current climate and limits the calculation load; however, it is unable to fully represent extreme conditions. There exist different approaches for creating a typical or reference weather year, and Herrera *et al*. [98] and Nik [9] have reviewed the major ones.

A common approach in resilience studies involves dividing weather into different states, for example two states of ‘normal’ and ‘adverse’, three states by adding an ‘extreme’ state, or multi-state models to represent a wider range of fluctuating weather conditions [99]. However, the available studies are mostly based on past climate and not considering future climate and its uncertainties. Two major challenges with extreme weather are estimating the rate of change and connecting that to the risk assessment [100]. Assessing and quantifying the climate resilience of energy systems requires proper connection of future climate, projected by climate models, to energy models. Moreover, as becoming climate resilient demands withstanding the plausible abnormal conditions, we need to know about the local climate with the hourly or sub-hourly temporal resolution and under many plausible future scenarios.

Future climate conditions are simulated by GCMs, adopting different initial conditions and forced by several forcing factors such as anthropogenic greenhouse gas (GHG) concentrations which depend on emission scenarios or concentration pathways (known as Representative Concentration Pathways or RCPs) developed by IPCC [24]. As a result of the coarse spatial resolution of GCMs, and recognized biases, their output cannot be directly used in energy system analyses [101]. Therefore, downscaling is needed to simulate local weather conditions. Two main approaches for downscaling GCMs are dynamical and statistical downscaling [9]. Dynamic downscaling often involves using an RCM, whereas statistical downscaling builds on the statistical relationship between large-scale climate and local climate established with the historical records. The morphing technique [102] is a widely used statistical approach, based on combing present-day observed weather data with GCM data. This approach only reflects changes in the average weather conditions and underestimates extremes. This is where dynamic downscaling can help, simulating weather data sets that are physically consistent across different variables and have suitable temporal and spatial resolutions [103]. Downscaling GCMs into different spatial resolutions results in different weather conditions. Moreover, the effects of urban and microclimate may induce considerable changes in the urban scale [104], which is hard, if not possible, to take into account by a conventional RCM. Nevertheless, observation of past climate, plus GCM and RCM simulations are important tools to provide needed weather data for energy system assessments.

**Figure 5.**
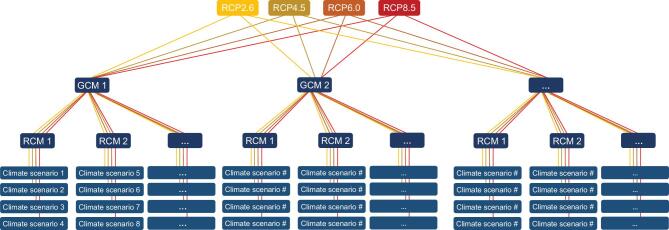
Global Climate Models (GCMs) are used to simulate future climate conditions. According to the Fifth Assessment Report (AR5) of IPCC, there are four Representative Concentration Pathways (RCPs), forcing GCMs to generate future climate projections: RCP2.6, RCP4.5, RCP6.0 and RCP8.5. Regional Climate Models (RCMs) are used to downscale GCMs dynamically with fine spatial and temporal resolution, enabling a physically consistent representation of climate variations and extremes. As a result of the existence of different RCPs, climate models and uncertainties, a thorough impact assessment of climate change should consider multiple climate scenarios. This increases the calculation load, especially considering the fine temporal and spatial resolutions that are required for climate resilience studies on the urban scale.

All in all, the synthesized weather data will be different depending on the selected GCM, RCM, emissions scenario, GHG concentration (or RCP) and the spatial resolution (Fig. 5 explains this for different RCPs, GCMs and RCMs). Consequently, it is not considered appropriate to rely on only a few numbers of climate scenarios. Moreover, relatively long periods (20–30 years) should be selected as the natural variability in the climate system is usually large. Therefore, short-term comparisons are not reliable [103]. This, together with the need for considering several climate scenarios, requires handling large data sets [9], which can become computationally expensive.

Access to representative and ready-to-use future weather files is another challenge for energy studies, which hopefully will fade away in the near future by the higher availability of future climate data sets and the increased interest in the energy sector. There are some ready-to-use weather data sets; however, they are mostly developed by extending the available approaches on the statistically downscaled GCM data (e.g. by applying the morphing technique). These data sets neglect future climate variations and anomalies and cannot represent extreme conditions. Therefore, they are not suitable for resilience studies. Fortunately, some approaches have been developed to consider extreme climatic conditions while keeping the calculation load affordable. For example, the weather generator of the UK Met Office, UKCP09, is trained using historical weather data and generates long-term weather data through random sampling. Schulz *et al*. [3] developed the Extreme Meteorological Year (XMY) using four combinations of extremes. Nik [9] developed a method for synthesizing representative future weather data sets out of RCMs, generating three data sets: Typical Downscaled Year (TDY), Extreme Cold Year (ECY) and Extreme Warm Year (EWY). The generated data sets include extreme conditions and overcome the challenge of future climate uncertainties by considering several climate scenarios, meanwhile keeping the calculation load limited. The application of the method has been compared with other available approaches and weather data sets [105] and verified against several types of simulations and impact assessments [9], including quantifying the impacts of climate change on urban energy systems [10].

Panteli and Mancarella [70] count 11 measures to enhance the long-term resilience of the energy network, one of which being the accurate estimation of the location and strength of extreme climate conditions. However, such an accurate estimation is almost impossible for future climate [9,64]. It is thus necessary to develop methods for assessing the climate resilience of energy systems that consider climate uncertainties and multiple future climate scenarios, as well as to account for high stochasticity and multi-dimensional impacts of weather (considering fine enough temporal and spatial resolutions).

## CLIMATE RESILIENCE OF URBAN ENERGY SYSTEMS: HOW TO PROCEED?

As discussed in the previous sections, the major challenges in assessing the climate resilience of urban energy systems are: (1) complex and multivariate energy flows in urban areas, (2) high dynamism and multi-scale variations of climate, (3) future climate uncertainties, (4) long-term future weather data sets and heavy calculation loads, (5) lack of standardized frameworks to communicate outputs of climate models to energy models or to generate representative future weather data sets for the energy and resilience studies, (6) ambiguous definitions/indicators for resilience, (7) lack of methods and frameworks to design and optimize energy systems for future climate and to assess climate resilience of urban energy systems.

**Figure 6.**
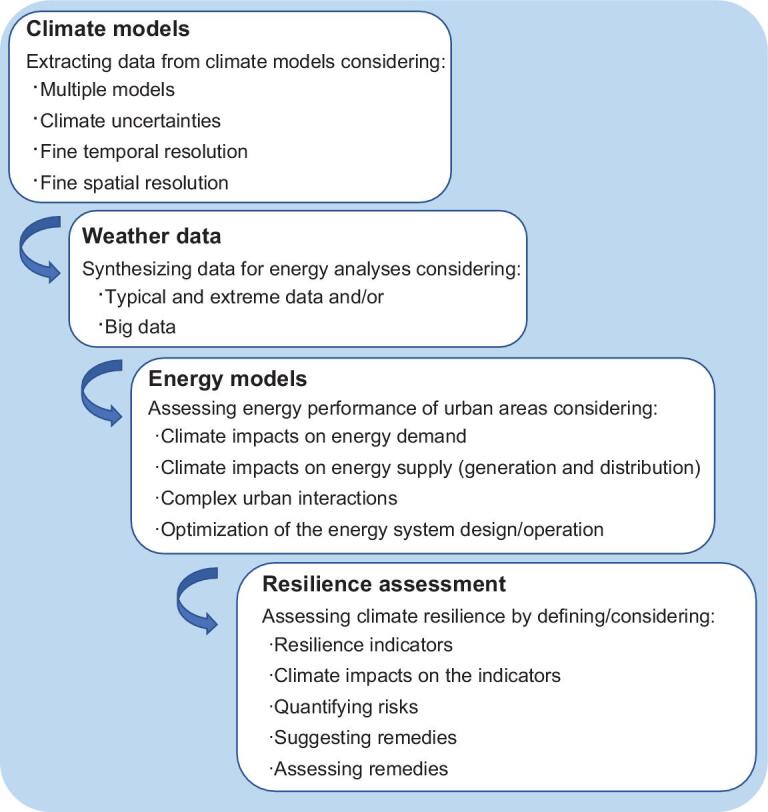
A general framework to assess the climate resilience of urban energy systems for future climate.

Field *et al*. [11] define climate change adaptation as the process of adjustment to the actual or anticipated climate to moderate harm and/or exploit beneficial opportunities. Climate resilience can be considered an important part of climate change adaptation which mostly deals with risky conditions and climate disasters [22]. Moving towards climate resilient urban energy systems, a design-focused vision needs to be adopted (together with operation-focused), especially when higher shares of renewable and distributed generation (DG) are integrated. Figure 6 provides a general framework for assessing the climate resilience of urban energy systems, illustrating different components that should be considered. Surely the adopted details, data and methods depend on the case study and availability of data.

Assessing the climate resilience of energy systems in the urban context requires consideration of a wide range of future climate projections and a framework that accounts for different components/levels (e.g. building, urban, generation, demand and supply, centralized and decentralized energy systems) and their complex interactions. Performing such an assessment requires one to:

- be flexible enough to readjust the resilience definition/criteria and decide on suitable performance criteria that serve the purpose,
- treat urban areas and energy systems as complex systems with complex interactions and account for impacts from the influencing factors and their associated uncertainties,
- select suitable platforms or frameworks that can assess climate resilience considering uncertainties and complex interactions, e.g. by adopting probabilistic approaches and Monto Carlo simulations,
- avoid single scenario approaches and consider multiple scenarios for the influencing factors (e.g. climate, urbanization, user behaviour, etc.) and their combinations,
- select a wide range of future climate scenarios to consider uncertainties and extremes with small probabilities and account for plausible future extreme events,
- apply updated and reliable climate scenarios, for example scenarios that are developed based on the most recent IPCC reports such as Coupled Model Intercomparison Project Phase 5 (CMIP5) and CMIP6,
- apply weather data with a fine enough spatial and temporal resolution, for example at least regional, and ideally urban, scale weather conditions with time resolution finer than 6 hours,
- develop/adopt suitable weather data sets and consider both long- and short-term variations of climate to develop the plan for a proper climate change adaptation,
- develop the energy system design and optimization methods/platforms further, to become faster in handling complex systems with large data sets. Methods powered by data analytics can be useful in this regard.

**Figure 7.**
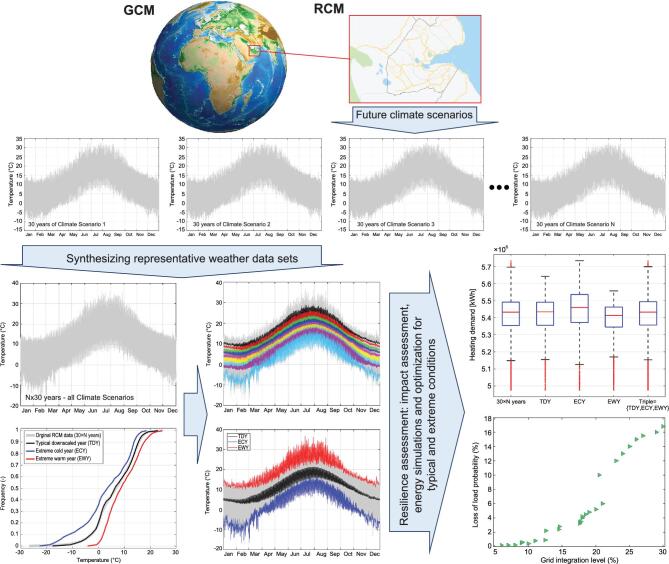
Assessing the climate resilience of urban energy systems demands consideration of several future climate scenarios (as well as scenarios to represent the variable conditions in urban areas). Representative weather data sets can be synthesized out of climate models to reduce the computation load without underestimating extreme conditions. For a system design, it is possible to combine several climate scenarios (to cover climate uncertainties) and synthesize typical and extreme scenarios (three weather data sets) as well as probabilistic sequences (e.g. seven data sets for the percentiles of 5–10–20–30–20–10–5%), to be used in energy simulations and to optimize urban energy systems (more details are available in Refs [9,10]).

It is important to synthesize and apply proper weather data sets that account for unpredictable but plausible extreme events, enabling climate resilient designs. For example, the method developed by Nik [9] has been extended for studying the climate flexibility and resilience of energy systems and their performance during extreme conditions [10] (check Fig. 7). To do so, instead of only relying on typical and extreme months, the weather data sets were synthesized to represent typical and extreme conditions per hour, by considering for example 390 scenarios (13 future climate scenarios for 30-year periods) (see Refs [9] and [10] for details). This enables addressing the extreme climatic conditions with the hourly temporal resolution while considering climate uncertainties. Moreover, the expected values of climate and energy variables were calculated (per hour) by assessing their cumulative distribution considering multiple climate scenarios. This resulted in several sets of pseudo-sequential time series of weather and energy data, representing the long-term variations of future climate in a meaningful way, which helps to address climate variations and uncertainties (see Ref. [10] for details). The developed methodology can also be applied to study climate resilience and perform predictive analyses using observed weather data and short-term weather forecasts.

## CONCLUDING REMARKS

A sustainable transition of the energy sector plays a major role in mitigating climate change. A successful transition is not possible without proper climate change adaptation and counting for climate uncertainties and extremes. Urban areas and their energy systems have great potential and responsibility for such a transition and climate change adaptation. Failing in that can lead to irreversible environmental conditions and heavy economic losses, while a resilient design can move the energy system towards higher flexibility and robustness.

It is necessary to thoroughly assess the transition pathways and quantify the risks and uncertainties. In this regard, climate resilience can be considered a critical part of climate change adaptation, mostly addressing extreme events and climate disasters. Therefore, having a climate resilient design of urban energy systems can lead to a resilient operation in the future, and consequently a safe transition. However, there are a number of major challenges in assessing the climate resilience of energy systems, with the most important one being a lack of proper frameworks and/or methods to transfer data and information from climate models to energy models.

Impacts of climate change, including extreme conditions (and the consequent uncertainties), on urban energy systems are mostly neglected in the energy system design phase, although there are very limited discussions on the operation phase. Most of the available resilience studies are focused on the energy supply, considering traditional and fossil fuel-based energy sources. The available approaches for assessing the resilience are mostly for the spatial scales larger than urban scale and focused on a single aspect. The major limitations include inadequate representation of probable future conditions because of the limited number of scenarios for future conditions and not considering extreme events, methodological limitations in the energy system design and optimization to address urban complexities, climate uncertainties, extreme weather events, as well as a lack of a clear definition and quantification of resilience and performance gap. All the aforementioned limitations make it difficult to understand the changes required in the superstructure of urban energy infrastructure. This may lead to either overconservative limits for renewable energy integration or even cascade failures and blackouts. The consequences are not limited to the energy sector, but even propagate to other interconnected infrastructures.

Assessing the climate resilience of urban energy systems is possible by considering a wide range of future climate projections and adopting a suitable framework to account for the different components that affect the energy flow in the urban scale, from generation to demand. The multivariate and multiscale variations as well as complex interactions and uncertainties that affect the energy flow in urban areas should be considered in the design and operation of energy systems and grids, especially those linked with extreme climate and high impact events. This requires further development of the energy modelling techniques and frameworks to consider climate change variations and quantify energy demand and renewable generation potential at the building, neighbourhood, district and urban scale. Moreover, it is needed to synthesize proper weather data sets that account for plausible extreme events. We suggest a general framework for assessing the climate resilience of urban energy systems, while the adopted details and methods can vary depending on the specific needs of a case. In practice, the developed framework can be used as an early warning system by being connected to weather forecasts. Before making decisions, relevant data from multiple sources needs to be gathered and analysed to create a valid representation of the upcoming conditions. Such an analysis is possible through data analytics and having an ever-updating model of the urban energy system or its digital twin.
